# Investigating the Structural Dynamics and Crack Propagation Behavior under Uniform and Non-Uniform Temperature Conditions

**DOI:** 10.3390/ma14227071

**Published:** 2021-11-21

**Authors:** Khangamlung Kamei, Muhammad A. Khan

**Affiliations:** School of Aerospace, Transport and Manufacturing, Cranfield University, Bedford MK43 0AL, UK; muhammad.a.khan@cranfield.ac.uk

**Keywords:** fatigue crack propagation, vibration analysis, crack depth measurement, modal parameters

## Abstract

The robustness and stability of the system depend on structural integrity. This stability is, however, compromised by aging, wear and tear, overloads, and environmental factors. A study of vibration and fatigue cracking for structural health monitoring is one of the core research areas in recent times. In this paper, the structural dynamics and fatigue crack propagation behavior when subjected to thermal and mechanical loads were studied. It investigates the modal parameters of uncracked and various cracked specimens under uniform and non-uniform temperature conditions. The analytical model was validated by experimental and numerical approaches. The analysis was evaluated by considering different heating rates to attain the required temperatures. The heating rates were controlled by a proportional-integral-derivative (PID) temperature controller. It showed that a slow heating rate required an ample amount of time but more accurate results than quick heating. This suggested that the heating rate can cause variation in the structural response, especially at elevated temperatures. A small variation in modal parameters was also observed when the applied uniform temperatures were changed to non-uniform temperatures. This study substantiates the fatigue crack propagation behavior of pre-seeded cracks. The results show that propagated cracking depends on applied temperatures and associated mass. The appearance of double crack fronts and multiple cracks were observed. The appearance of multiple cracks seems to be due to the selection of the pre-seeded crack shape. Hence, the real cracks and pre-seeded cracks are distinct and need careful consideration in fatigue crack propagation analysis.

## 1. Introduction

Engineering structures and components experience fatigue and failure during operations. This failure is due to many reasons, such as wear and tear, cycles of loads, working environments, crack occurrence and propagation, etc. Researchers considered that early detection is the solution to prevent catastrophic damage. Modal analysis is one of the common techniques used to study structure health monitoring [1,2,3]. This paper aims to study the modal and crack propagation behavior of a beam subjected to thermal and mechanical loads.

Generally, the vibration of the components impacts the durability and reliability of the system. Warminska et al. [4] outlined that structural dynamics were driven by thermal distribution. In a similar study, Yang and Shen [5] stated that vibration response was determined by the thermal effect, boundary conditions, and material composition. Shen and Wang [6] identified temperature as impacting the vibration frequency and marginal influence on the nonlinear-to linear frequency ratio. Kitipornchai et al. [7] studied random vibration and noticed it to be affected by temperatures. Fatigue due to vibration is also studied based on applications [8,9,10]. Vibration-based studies examined the dynamic response of beams and damage quantification approaches for metallic and non-metallic materials [11,12,13,14]. The vibration analysis method was often used to extract defects and faults in real applications such as an exhaust manifold [15], a turbine blade and rotor [16,17], servo-hydraulics [18], a jet engine [19], etc. The mechanical system was disturbed by coupled loads. The changes in applied thermal and mechanical loads alter the fundamental frequencies of the system [20,21]. The thermal effect on structures was studied in different scenarios [22,23,24,25,26,27,28,29]. Techniques were developed to predict the high-frequency response of beams, explore the natural thermal vibration, and study the mechanical fatigue of metallic beams. The researchers noticed that the applied thermal load generates thermal stresses and changes in mechanical properties. Thermal vibration was caused by thermally induced expansion. It was also found that the interatomic bond length increases with increasing temperatures [23]. Recent review papers on the dynamic response of the system at elevated temperatures can be found in the literature [1,2,30,31,32,33].

Even though there are various studies on structure dynamics, the existing research has not considered the heating duration and non-uniform temperatures conditions which could potentially affect the structural dynamics. In this study, the modal parameters and crack propagation behavior of a cantilever beam subjected to thermal and mechanical loads were evaluated. This beam was selected because many aircraft wings and turbine blades operate akin to a cantilever beam. This research considered three heating rates to examine the effect of heating duration on structural instabilities. Ramping of 2 °C/min was assumed as a slow heating rate, 5 °C/min as moderate, and 8 °C/min as high. This variation in ramping temperature to achieve the required temperature allowed the beam to be exposed to the heat for different durations, even for the same temperature. The evaluation of the modal response and fatigue crack propagation analysis was conducted separately for uniform and non-uniform temperatures. A pre-seeded crack of rectangular shape was selected to evaluate the crack propagation in this study.

The finite element method (FEM) and dual boundary element method (DBEM) are further techniques used to analyze the structural dynamics. FEM is commonly used to study large structures. One of the disadvantages of FEM is modelling cracks in FEM comprises re-meshing of the element particularly during crack growth [34,35]. Moreover, DBEM simplifies the step of the remeshing process and acquires more accurate results by capturing the stress field at the crack front [36,37]. Recently, a coupled FEM-DBEM approach was proposed based on the superposition principle to analyze crack scenarios [38,39]. This coupled procedure has the advantages of minimizing the runtimes, and is able to predict the stress intensity factor, crack growth and its crack paths with accuracy. However, this paper is more focused on the analytical and experimental factors for crack propagation analysis. However, we used numerical modelling for comparative studies on modal parameters, thermal distribution and stress–strain distribution only, and hence present our results.

The paper proceeds with an analytical formulation to estimate the modal parameters subjected to thermal and mechanical loads. This formulation considered the effective length of the beam due to thermal expansion along with the accelerometer mass in a fixed-free boundary condition. Next, a linear temperature distribution approximation along the beam is presented. The experimental method and numerical analysis are then described on the basis of a test performed in uniform and non-uniform temperature conditions. In the Results and Discussion section, the thermal properties of the experimental metal Al 2024-T3 and thermal and elastic strain intensity analysis are discussed. The comparison of the modal behavior of a beam under uniform and non-uniform temperatures conditions is also reported. The last section presents the crack propagation behavior when subjected to thermal and mechanical loads.

## 2. Material and Methods

### 2.1. Analytical Formulation

The study of the structural dynamics of cantilever beams is familiar, because many engineering applications, particularly aircrafts and turbines, operate akin to a cantilever beam. In this formulation, the Bernoulli–Euler beam theory with a uniform cross-section in fixed-free boundary conditions is considered. The vibration force F(x,t) and the accelerometer with its mass m is mounted at the free end of the beam. Generally, the original length of the beam changes due to the applied temperature. Therefore, the effective length of the beam $L_{e}$, which is the original length with its thermal expansion, is considered for this analysis. The beam is assumed in one-dimensional longitudinal expansion only because the beam width *B* and thickness *h* are insignificant compared to its length. The equation of the beam can be expressed as:

(1) $$F\left(x,t\right)=EI\frac{\partial^{4}u}{\partial x^{4}}\left(x,t\right)+\left(\rho A+m\right)\frac{\partial^{2}u}{\partial t^{2}}\left(x,t\right)$$

where u represents the transverse displacement, ρ is the mass density of the beam, and A is the cross-sectional area. The applied force is zero for free bending vibration, and consequently, the equation is reduced as:

(2) $$\frac{EI}{\left(\rho A+m\right)}\left(\frac{\partial^{4}u\left(x,t\right)}{\partial x^{4}}\right)+\frac{\partial^{2}u\left(x,t\right)}{\partial t^{2}}=0$$

The solution of Equation (2) can be obtained through a separation of dependent variable method expressed as:

(3) u(x,t)=U(x)V(t)

Thus, the free vibration equation of the beam can be deduced in the form of a separable variable method as:

(4) $$-\frac{EI}{\left(\rho A+m\right)}\frac{\left(\frac{d^{4}U\left(x\right)}{dx^{4}}\right)}{U\left(x\right)}=\frac{\frac{d^{2}V\left(t\right)}{dt^{2}}}{V\left(t\right)}$$

Let $\omega^{2}$ be constant, then

(5) $$-\omega^{2}=-\frac{EI}{\left(\rho A+m\right)}\frac{\left(\frac{d^{4}U\left(x\right)}{dx^{4}}\right)}{U\left(x\right)}=\frac{\frac{d^{2}V\left(t\right)}{dt^{2}}}{V\left(t\right)}$$

Separating the time and spatial variable, respectively, we have:

(6) $$\frac{d^{2}V\left(t\right)}{dt^{2}}+\omega^{2}V\left(t\right)=0$$

(7) $$\left(\frac{d^{4}U\left(x\right)}{dx^{4}}\right)-\omega^{2}\frac{\left(\rho A+m\right)U\left(x\right)}{EI}=0$$

Solving the free vibration equation of the beam as discussed in our previous paper [40], we obtained the fundamental frequency of the beam expressed as:

(8) $$f=\frac{\omega }{2\pi }=\frac{{\left(\beta_{n}L_{e}\right)}^{2}}{2\pi }\sqrt{\frac{EI}{\left(\rho A+m\right){L_{e}}^{4}}}$$

The modulus E is a temperature-dependent property that varies with temperature. The modulus decreases with increasing thermal energy along the beam. This decrease is assumed as being linear with temperature rise. The approximate relationship [41] is given as:

(9) $$E\left(T\right)=E_{o}\left(1-\varphi \frac{T}{T_{o}}\right)$$

where φ=0.3 is the proportional constant, $T_{o}$ is the melting temperature, T is the temperature at the measured value of  E, and $E_{o}$ is the modulus at 20 °C. When the beam is long, such that it has different temperature zones across the beam length, the effective or the equivalent modulus of the beam can be calculated by taking the average of the modulus. Considering that the effective modulus $E_{e}$ is more applicable when the cross-section area of the beam is non-uniform, the fundamental frequency of the beam due to linear temperature distribution can be obtained as:

(10) $$f=\frac{\omega }{2\pi }=\frac{{\left(\beta_{n}L_{e}\right)}^{2}}{2\pi }\sqrt{\frac{E_{e}I}{\left(\rho A+m\right){L_{e}}^{4}}}$$

The modifications in the fundamental frequency due to the crack propagation on the beam can be found as [42]:

(11) $$\Delta \omega_{nc}=\frac{\Delta Q}{2Q}\omega_{n}$$

where Q is the total deformation energy due to the beam deflection and ΔQ is the change in the deformation energy due to crack formation, represented as:

(12) $$\Delta Q=\frac{M^{2}}{2\chi_{t}}$$

where $\chi_{t}$ is the stiffness of the crack beam represented by Equation (13)

(13) $$\chi_{t}=\frac{Bh^{2}E_{e}}{72\pi F\left(\frac{t_{c}}{H}\right)}$$

where $F\left(\frac{t_{c}}{H}\right)=0.638{\left(\frac{t_{c}}{H}\right)}^{2}-1.035{\left(\frac{t_{c}}{H}\right)}^{3}+3.720{\left(\frac{t_{c}}{H}\right)}^{4}-5.177{\left(\frac{t_{c}}{H}\right)}^{5}+7.553{\left(\frac{t_{c}}{H}\right)}^{6}-7.332{\left(\frac{t_{c}}{H}\right)}^{7}+2.491{\left(\frac{t_{c}}{H}\right)}^{8}$ is the crack function and $t_{c}$ is the crack depth.

Thus, the new natural frequency of crack beam can be computed from Equation (14):

(14) $$\omega_{nc}=\left(1-\frac{\Delta Q}{2Q}\right)\omega_{n}$$

The amplitude of the beam was estimated using Equation (3). The modal amplitude is dependent on the respective natural frequencies of crack and non-damaged beam. The natural frequencies of the beam at different temperatures were analyzed using Equation (10). The change in the frequencies due to crack formation was computed using Equation (14).

### 2.2. Linear Temperature Distribution Approximation

**Figure 1:**
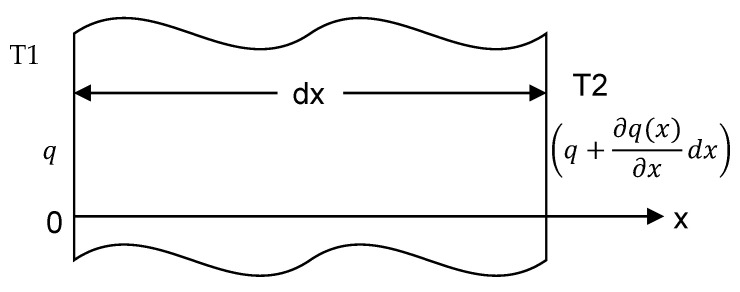
Heat flow by conduction.

Aircraft wings and turbine blades operate akin to a cantilever beam, and the temperature distribution is not uniform, but instead resembles a type of linear temperature distribution. This beam can be treated as one-dimensional because the thickness of the blades is thin as compared to its length. In such a case, we can use the one-dimensional law. We expressed the heat conduction equation based on Fourier’s law of the beam in Equation (15). Figure 1 shows the heat flow by conduction. We can write the equilibrium energy as Equation (16).

(15) $$q=-kA\frac{\partial T}{\partial x}$$

(16) $$q-\left(q+\frac{\partial q\left(x\right)}{\partial x}dx\right)=0$$

By solving the equation, we can find that $\left(-kA\frac{\partial^{2}T}{\partial x^{2}}\;\right)=0$. If the cross-section area and thermal conductivity are constant, we find that $\left(\frac{\partial^{2}T}{\partial x^{2}}\;\right)=0$. Integrating, we get $\frac{\partial T}{\partial x}=C_{1}$, Integrating again, we obtain

(17) $$T=C_{1}x+C_{2}$$

where $C_{1}$ and $C_{2}$ are integration constants, which can be found from the given boundary conditions as $T\left(0\right)=T_{1}\;at\;x=0$ and $T\left(L_{e}\right)=T_{2}\;at\;x=L_{e}$.

Hence, $T_{1}=C_{2}\;and\;T_{2}=C_{1}L_{e}+T_{1}$.

Therefore, the linear temperature distribution at any point on the beam can be found as:

(18) $$T\left(x\right)=\left(\frac{T_{2}-T_{1}}{L_{e}}\right)x+T_{1}$$

### 2.3. Experimental Methods

**Figure 2:**
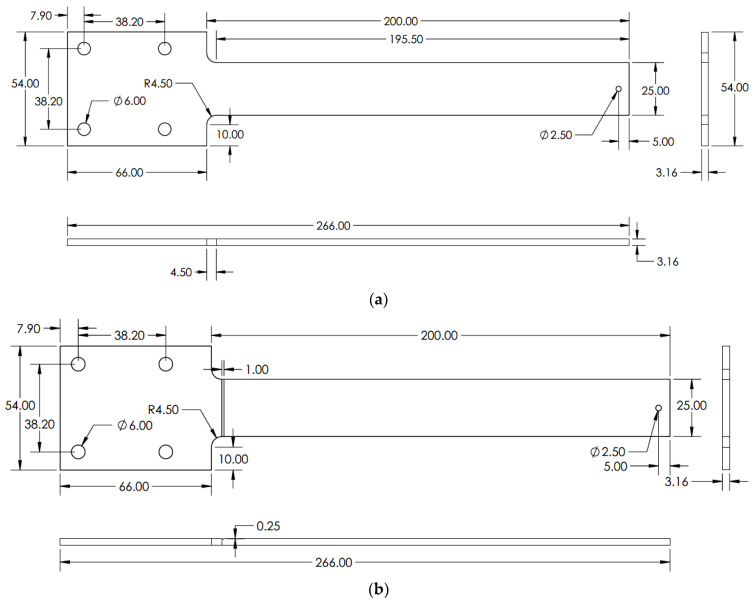
Specimen geometry dimensions in mm (**a**) without crack (**b**) with crack.

Aluminium 2024-T3 was selected for this research. Three types of specimens: without crack, with crack, and propagating cracks were chosen for the experiment. The crack specimens were classified into three crack depths: 0.25, 0.5 and 1 mm, each with a rectangular shape. This predefined crack depth was chosen to test the beam at various crack conditions under thermal and mechanical loads. The crack location was the same for of all the experiment specimens, which were 4.5 mm away from the fixed end. This crack location was considered because of the maximum stress concentration in the fillet region of the geometry. The geometry and its dimensions are shown in Figure 2.

**Figure 3:**
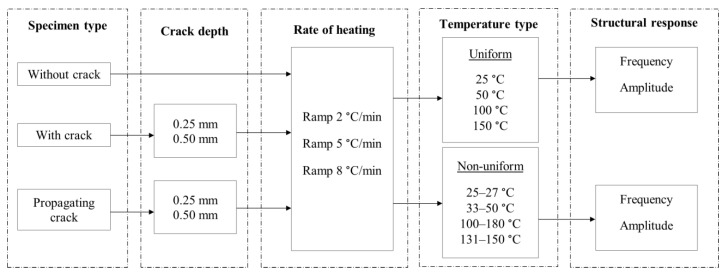
Detail of the experiments.

**Figure 4:**
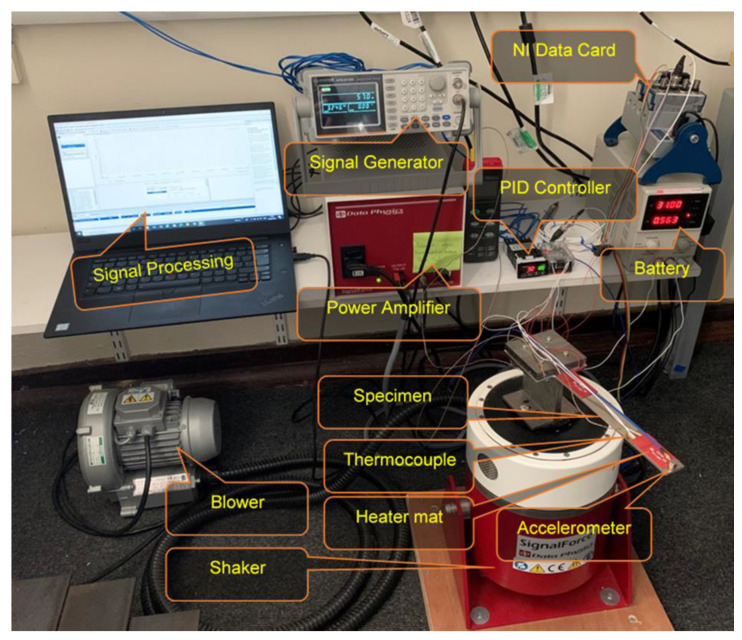
Experiment set-up.

The specimens were manufactured to have accurate dimensions using a computer numerical control machine. This experimental arrangement was considered to access the modal response of the beam under uniform and non-uniform temperatures at a maximum temperature of 200 °C. The beam response was evaluated at three heating rates, namely 2, 5 and 8 °C/min. The first heating rate was assumed as a slow heating rate, while the others were assumed as moderate and high, respectively. These rates were chosen to test the influence of heating duration on modal parameters. This controlled rate of heating was achieved by using a proportional-integral-derivative (PID) temperature controller manufactured by Omega engineering, Manchester, UK. The detail of the experimental scheme is shown in Figure 3, and the experimental setup is shown in Figure 4. For uniform temperature, the heat was supplied in both heating mats. However, the heat was supplied only at one heating mat for non-uniform temperature, i.e., a linear temperature distribution. Thereby, we were able to achieve the linear temperature distribution across the beam. 

### 2.4. Numerical Analysis

The numerical simulation to analyze the modal parameters was conducted using the ANSYS©2019 R2 workbench (Ansys, Inc. Canonsburg, PA, USA). The geometry of the specimen was drawn from the build-in design modeler. The model was built in a fixed-free boundary condition. The sinusoidal load with an amplitude of 2 mm was enforced as a mechanical load at the fixed end. The thermal loads were provided in a steady-state condition. The thermal properties of the material, such as the elastic modulus, coefficient of thermal expansion, and thermal conductivity were incorporated into the model at respective temperatures. The modal analysis module was utilized to evaluate the modal frequency of the beam for healthy and pre-seeded crack specimens separately. The harmonic module was used to analyze the modal amplitude of the beam. The meshed density of this study was 3 mm, with 8833 nodes and 1728 elements. This evaluation of modal parameters was conducted separately for with crack and without crack conditions at various temperatures.

## 3. Results and Discussions

### 3.1. Thermal Properties of Al 2024-T3

**Figure 5:**
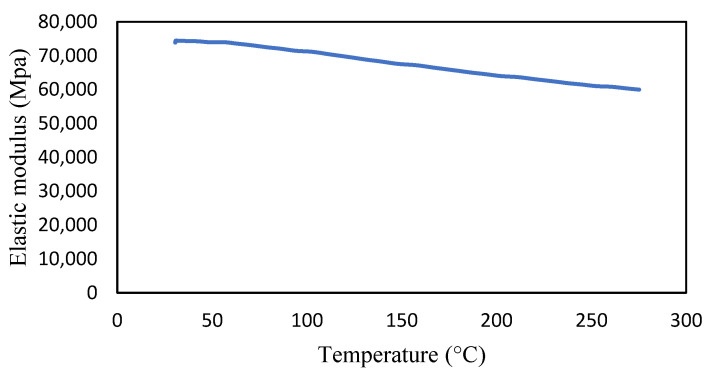
Elastic modulus of Al 2424-T3.

**Figure 6:**
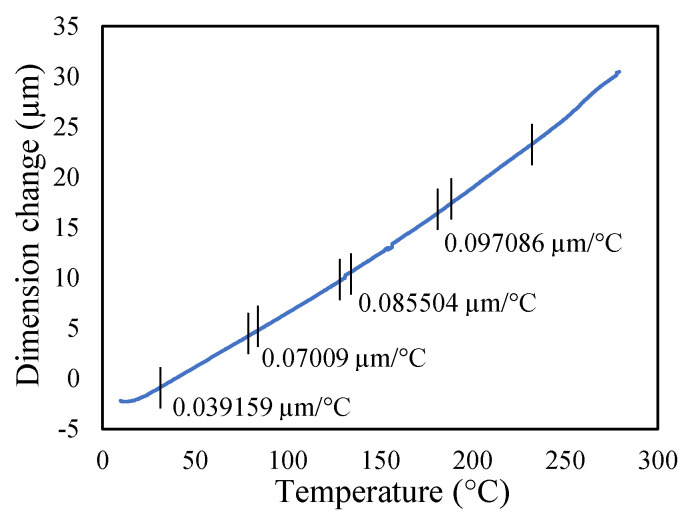
Thermal expansion of healthy specimen.

**Figure 7:**
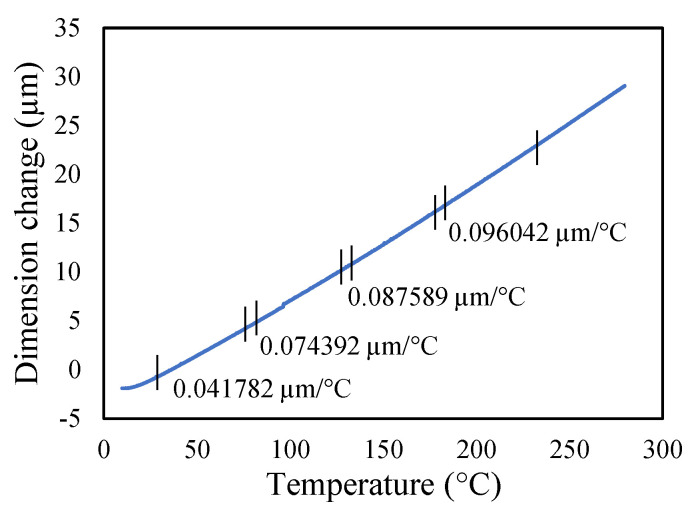
Thermal expansion of 0.25 mm crack depth specimen.

**Figure 8:**
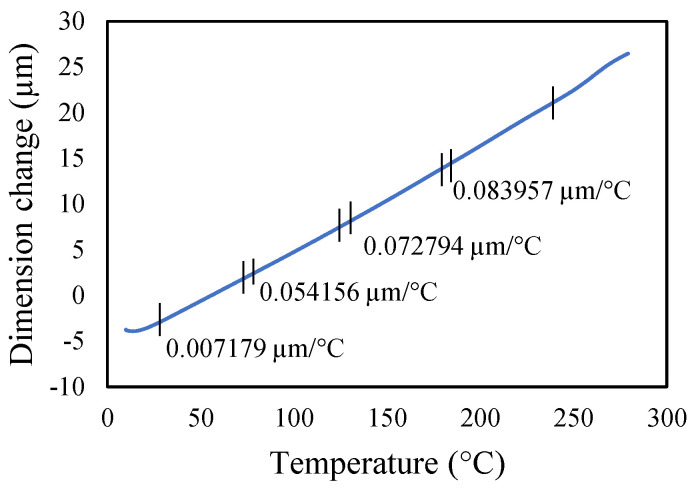
Thermal expansion of 0.5 mm crack depth specimen.

**Figure 9:**
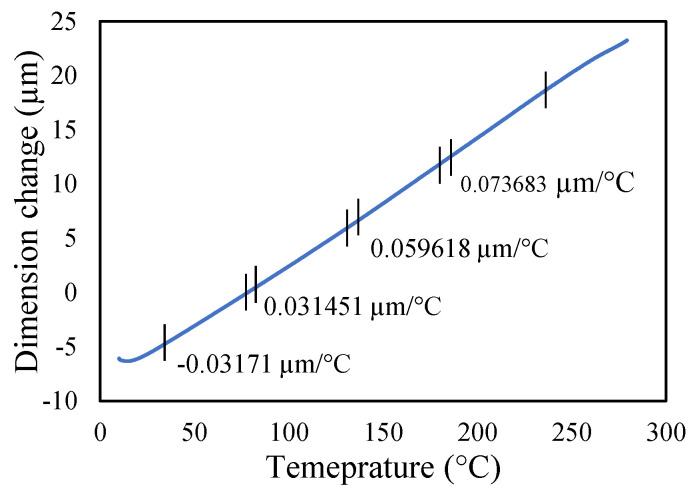
Thermal expansion of 1 mm crack depth specimen.

Aluminium 2024-T3 is selected for this research. The thermal properties of Al 2024-T3 were tested for various temperatures in the dynamic mechanical analyzer (DMA) and thermal-mechanical analyzer (TMA). The macro-expansion probe was used for TMA results; however, a single cantilever clamp was used for elastic modulus DMA results. The elastic modulus is sensitive to temperatures, as shown in Figure 5. The modulus decreases linearly with the temperature rise. The tests were conducted with a temperature ramp of 2 °C/min to raise the temperature from 30 to 250 °C. The coefficient of thermal expansion of healthy specimen is shown in Figure 6, and crack specimens of 0.25, 0.5, and 1 mm crack depth are shown in Figure 7, Figure 8 and Figure 9, respectively. The results show that thermal expansion increases linearly with the temperature rise. It also shown that changes in temperature and crack depths have small changes in dimension per degree Celsius as depicted in the figures. This suggests that the coefficient of thermal expansion varies per degree Celsius and also concerning crack depth.

The thermal expansions were measured without crack and with crack conditions. It was noticed that the coefficient of thermal expansion is increased from 0.039 to 0.097 µm/°C in the case of without cracks. However, in 0.25 mm crack depth it increases from 0.041 to 0.096 µm/°C while, in 0.5 mm it rises from 0.0071 to 0.083957 µm/°C and 0.1 mm crack depths start from −0.031 to 0.073 µm/°C. Therefore, the coefficient of thermal expansion is not uniform in all cases. Comparatively, the greater temperature has higher thermal expansion per degree Celsius than lower temperature. This indicates that the thermal expansion increases linearly with an increase in temperature, but with a different rate and crack conditions.

### 3.2. Thermal and Elastic Strain Intensity Analysis

The applied temperature is one of the factors that affect the modal response of the beam. In this study, two types of temperatures, uniform and non-uniform temperature loads, were employed to evaluate the effect on modal parameters and stress and strain intensity. The evaluations were executed separately for both temperatures and different cracked and uncracked specimens. In uniform temperatures, the temperature across the beam was maintained at the same temperatures. However, in non-uniform temperatures, the two ends of the beam were maintained at different temperatures, as described in the details of the experiments. The numerical analysis showed that the temperatures were linearly distributed across the beam in the case of non-uniform temperatures. The heat flow was from the high-temperature region to the low-temperature zone. In this study, all the higher temperatures were maintained at the free end, while lower temperatures were maintained at the fixed end. It was noticed that heat flux was concentrated more at the crack region, which was the fillet zone of the beam.

**Figure 10:**
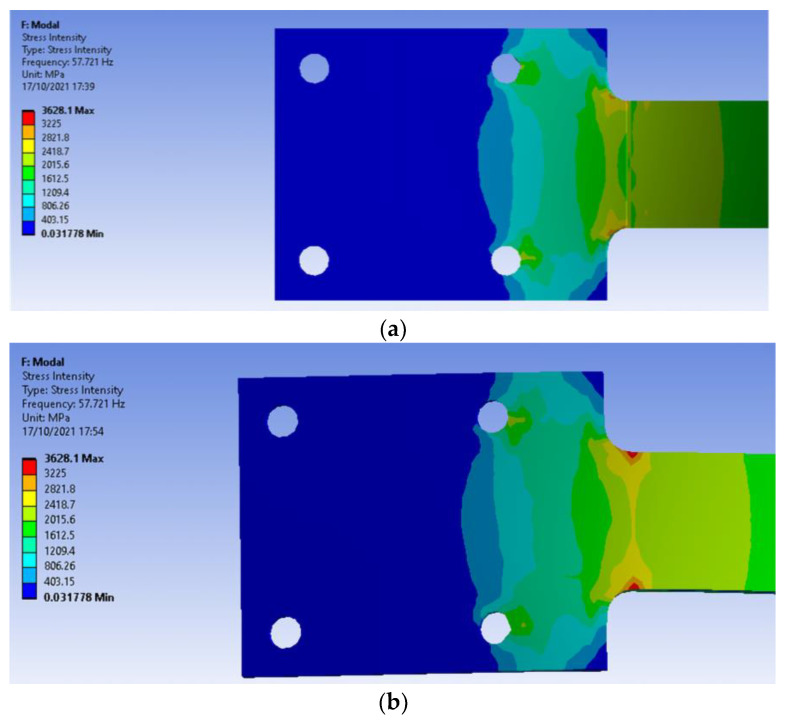
Stress intensity of 0.25 mm crack depth at 100–180 °C. (**a**) Top view, (**b**) Bottom view.

The assessment of stress and strain intensity was conducted for all types of specimens in both uniform and non-uniform temperatures distribution. It was observed that there was a stress concentration in the crack zone than in any other area of the beam. Moreover, the stress and strain intensity were more concentrated at the back of the crack region than in the crack area, as shown in Figure 10. It was also noticed that the stress concentration for the uncracked specimen was in the fillet region of the specimen. It was seen that stress and strain were produced more when the temperature increases. There was an augmentation of stress and strain intensity in the crack tip region when the crack depth increases. Hence, crack depth and applied thermal influence the stress and strain intensity.

### 3.3. Modal Behavior of a Beam under Uniform and Non-Uniform Temperature

**Figure 11:**
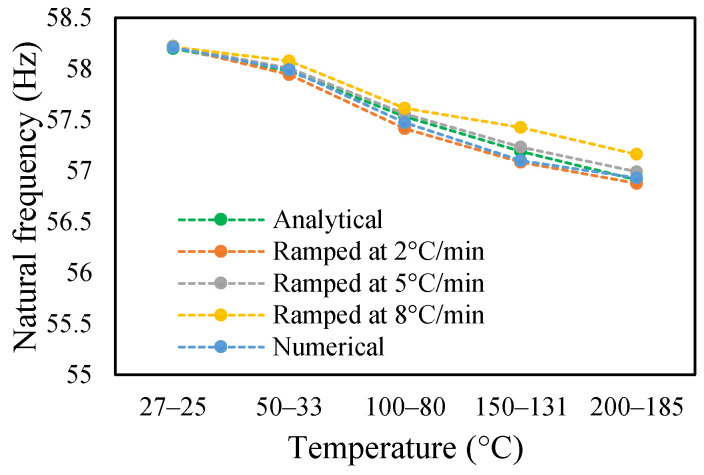
Natural frequency of healthy specimen in non-uniform temperature.

**Figure 12:**
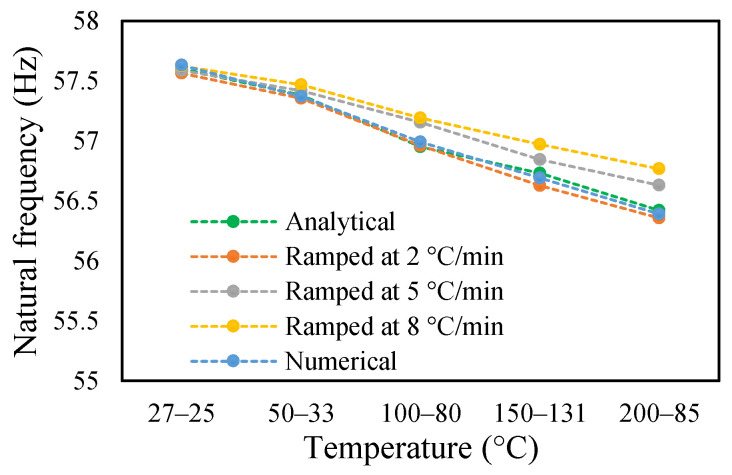
Natural frequency of 0.25 mm crack depth in non-uniform temperature.

**Figure 13:**
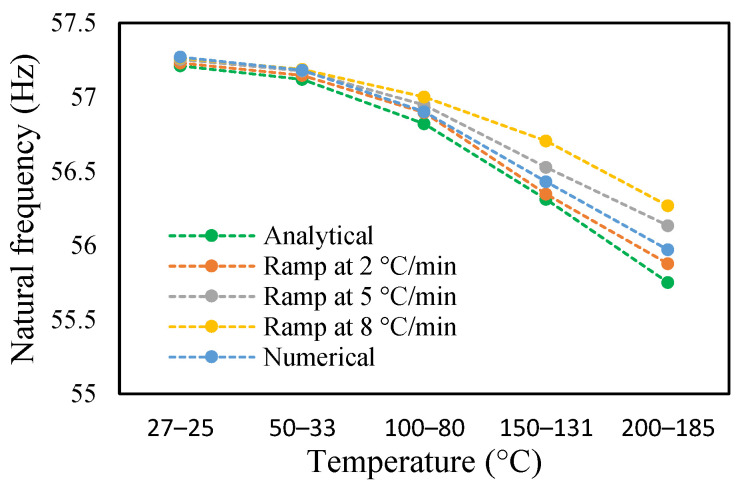
Natural frequency of 0.5 mm crack depth in non-uniform temperature.

**Figure 14:**
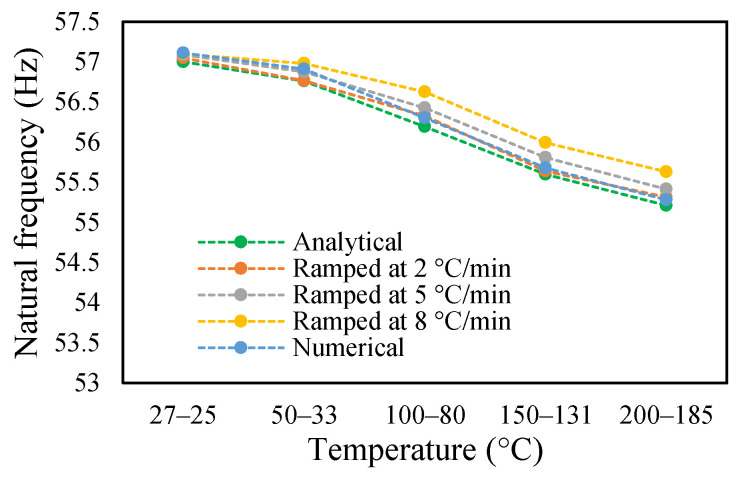
Natural frequency of 1 mm crack depth in non-uniform temperature.

This research work studies the modal behavior of the cantilever beam when subjected to thermal and mechanical loads. The evaluation of the modal parameters was implemented in a uniform and non-uniform temperature distribution conditions. The modal behavior of the beam was examined at three heating rates, as discussed in a previous paper [40]. The modal characteristic of the beam in uniform temperature was presented in a previous paper [40]. This paper analyzed the beam response under non-uniform temperature conditions and comparison of the modal dynamics in both temperatures. The natural frequencies of the healthy and crack specimens were evaluated. The decrease in modal parameters with the temperature rise was seen in the case of uniform temperature. The decreasing trend of fundamental frequency for the healthy specimen is shown in Figure 11. The natural frequencies of crack specimens with crack depths of 0.25, 0.5 and 1 mm are presented in Figure 12, Figure 13 and Figure 14, respectively. It was seen that heating rates impacted the modal frequencies, as depicted in the figures. Moreover, this heating effect was negligible at low temperatures but considerable at high temperatures.

**Figure 15:**
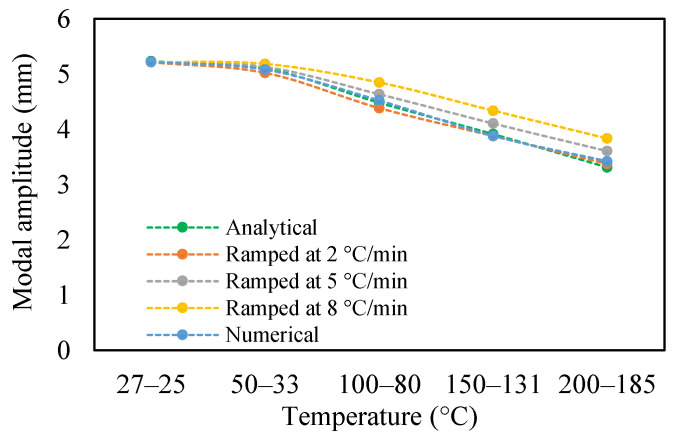
Modal amplitude of healthy specimen in non-uniform temperature.

**Figure 16:**
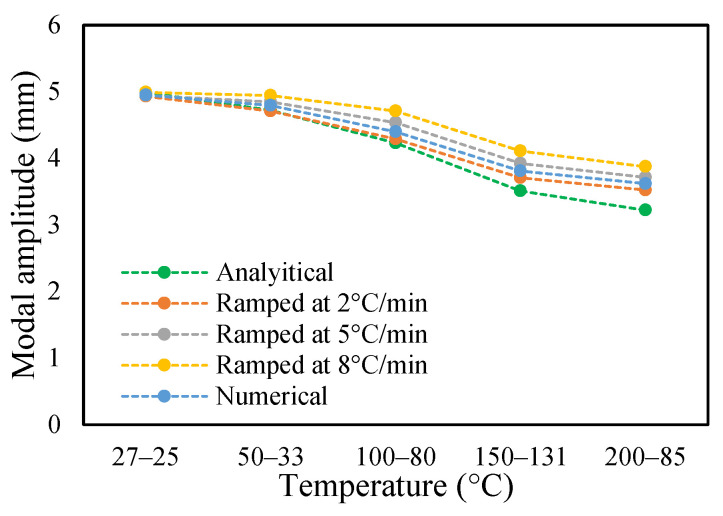
Modal amplitude of 0.25 mm crack depth in non-uniform temperature.

**Figure 17:**
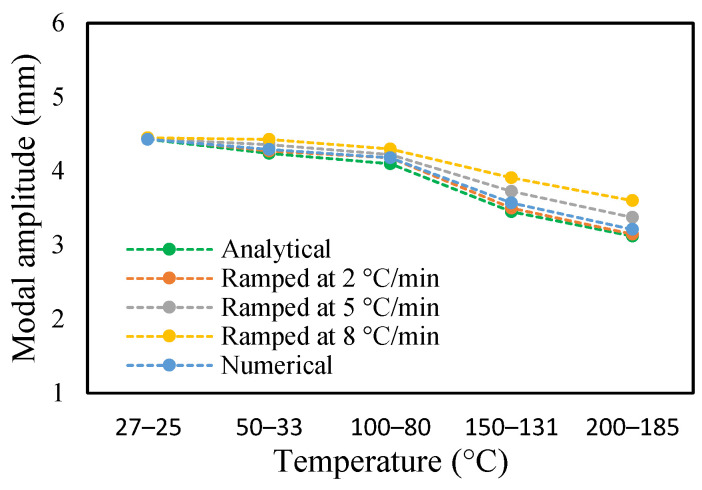
Modal amplitude of 0.5 mm crack depth in non-uniform temperature.

**Figure 18:**
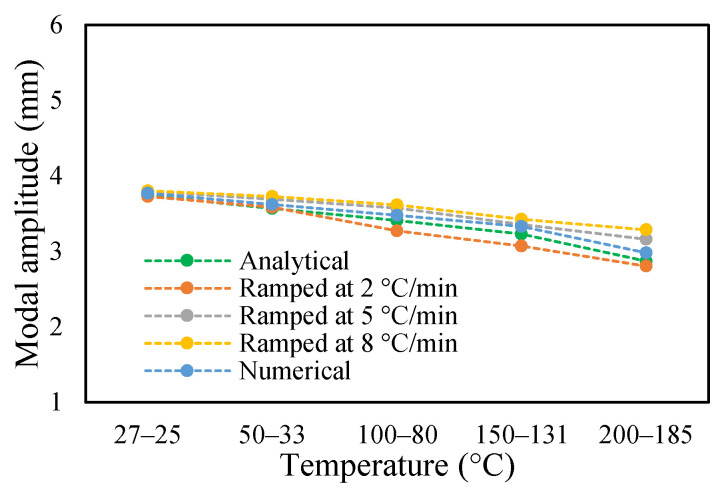
Modal amplitude of 1 mm crack depth in non-uniform temperature.

The change in fundamental frequencies for crack depths was also visible. The frequencies decrease when the crack depth increases. This drop in frequencies was steadier at low temperatures and crack depths. However, this tends to progress rapidly when the crack depth and temperature rise. However, this frequency behavior is limited, as it becomes steadier once it reaches certain crack depths, as represented in Figure 13 and Figure 14. The modal amplitudes of the beam when subjected to thermal and mechanical loads for uncracked samples and samples with crack depths of 0.25, 0.5 and 1 mm are presented in Figure 15, Figure 16, Figure 17 and Figure 18, respectively. The amplitude drop corresponded to the frequencies. It decreases with the decrease in frequencies as the temperatures rise. Similarly, the heating effect affected it. The ramp in temperature of 2 °C/min showed the lowest amplitude compared to the ramp of 5 °C/min and 8 °C/min. This signifies that heating at a lower rate accommodates more heat into the beam, and thereby the beam becomes less stiff. However, this effect was insignificant at low temperatures and was more considerable at high temperatures.

**Figure 19:**
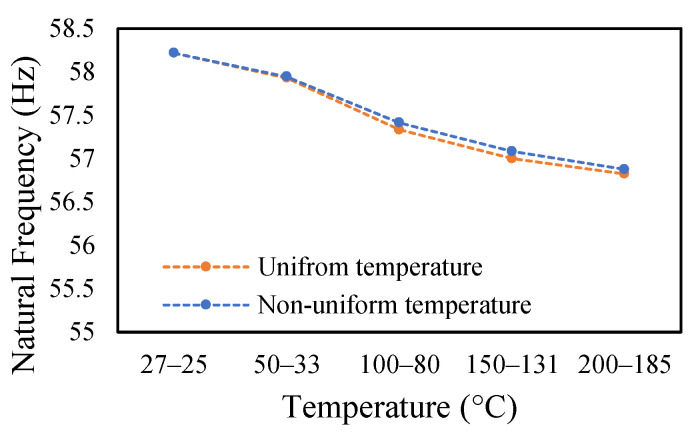
Comparison of natural frequency of undamage specimen ramped at 2 °C/min.

**Figure 20:**
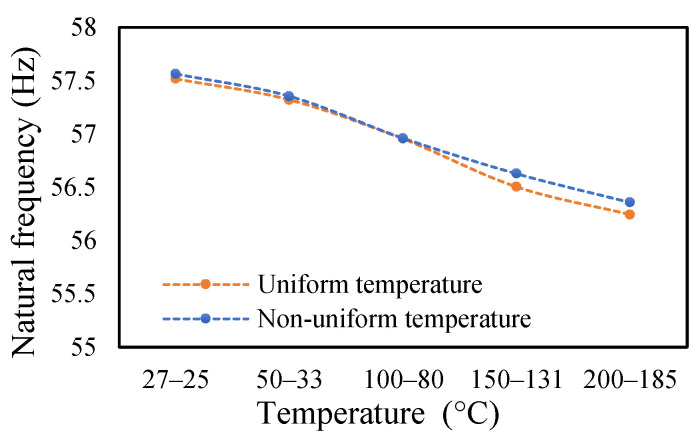
Comparison of natural frequency of 0.25 mm crack depth ramped at 2 °C/min.

**Figure 21:**
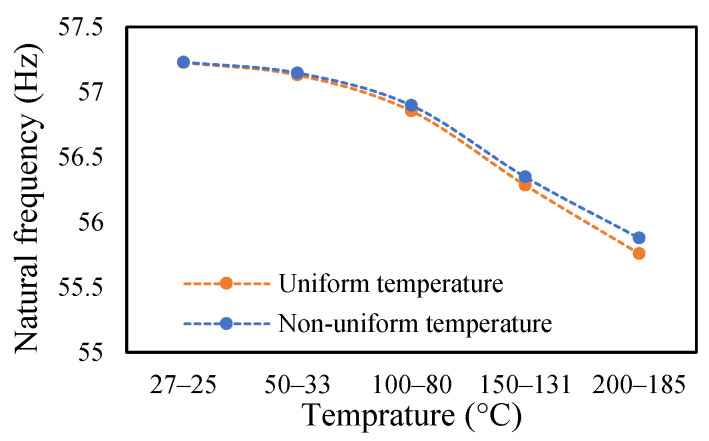
Comparison of natural frequency of 0.5 mm crack depth ramped at 2 °C/min.

**Figure 22:**
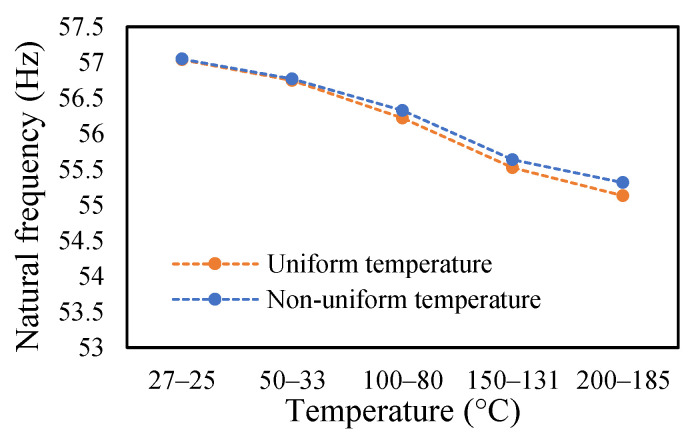
Comparison of natural frequency of 1 mm crack depth ramped at 2 °C/min.

In this study, the modal parameters of the cantilever beam were compared for both uniform and non-uniform temperatures. The results show that the modal parameters vary for both the temperatures conditions. It was noticed that the modal parameters were higher under non-uniform temperatures than under uniform temperatures. Even though there was a small variation in their responses, it was still noticeable. This small difference is due to the small temperature ranges. Their dissimilarities were minimal at low temperatures and visible at higher temperatures. The comparison of the fundamental frequencies for healthy and various crack depths specimens is shown in Figure 19, Figure 20, Figure 21 and Figure 22. Similarly, the modal amplitude of the cantilever beam varies for both temperatures. The results show that the amplitude differences were obvious only at high temperatures.

**Figure 23:**
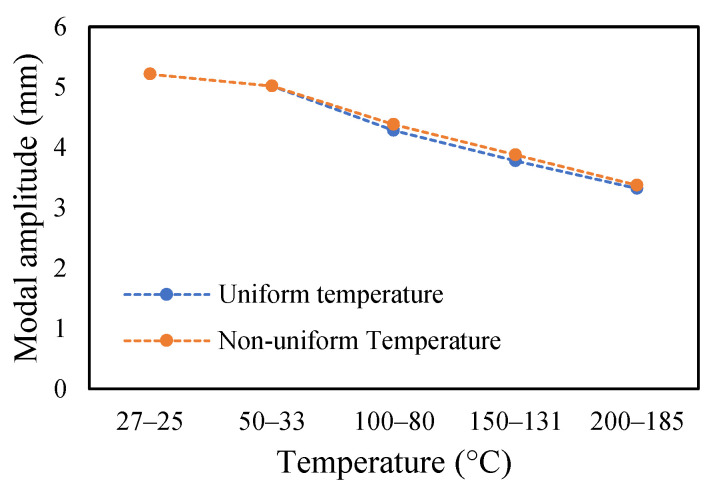
Comparison of modal amplitude of undamaged specimen ramped at 2 °C/min.

**Figure 24:**
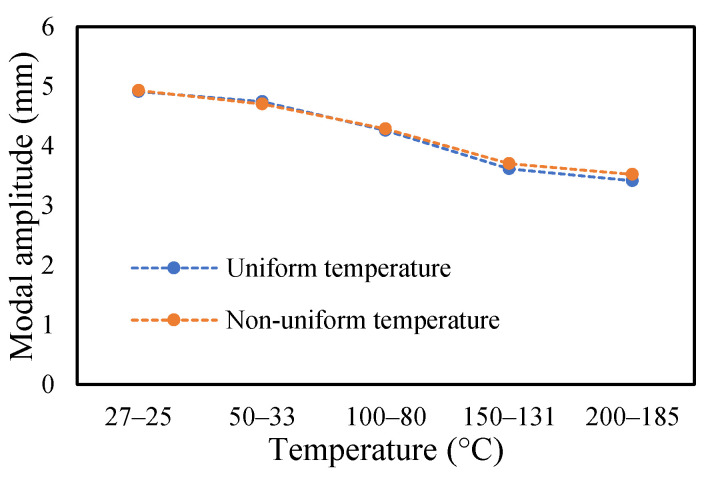
Comparison of modal amplitude of 0.25 mm crack depth ramped at 2 °C/min.

**Figure 25:**
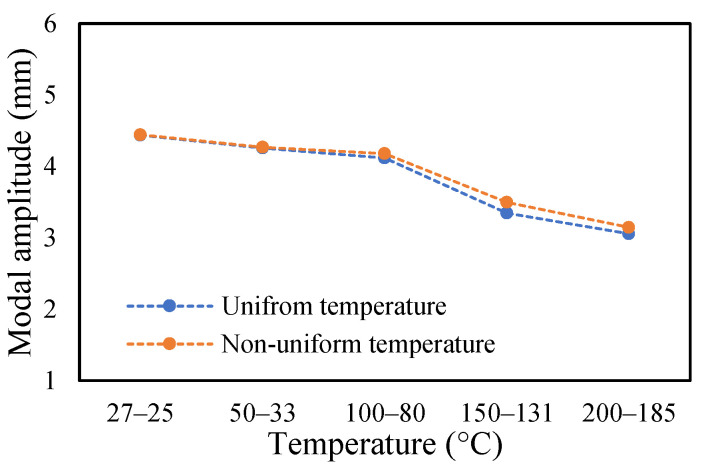
Comparison of modal amplitude of 0.5 mm crack depth ramped at 2 °C/min.

**Figure 26:**
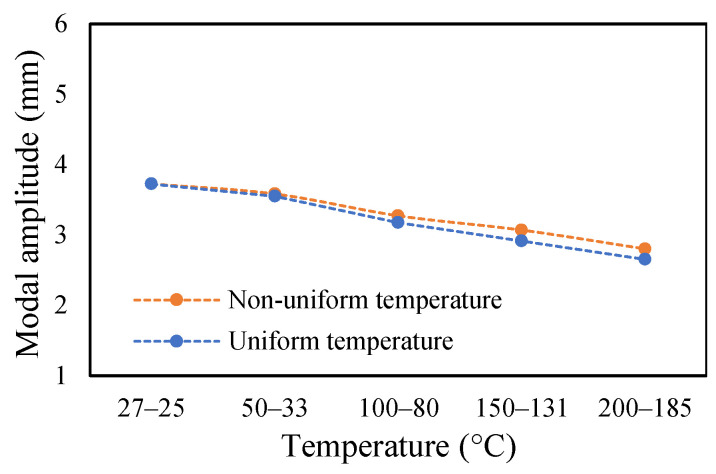
Comparison of modal amplitude of 1 mm crack depth ramped at 2 °C/min.

**Figure 27:**
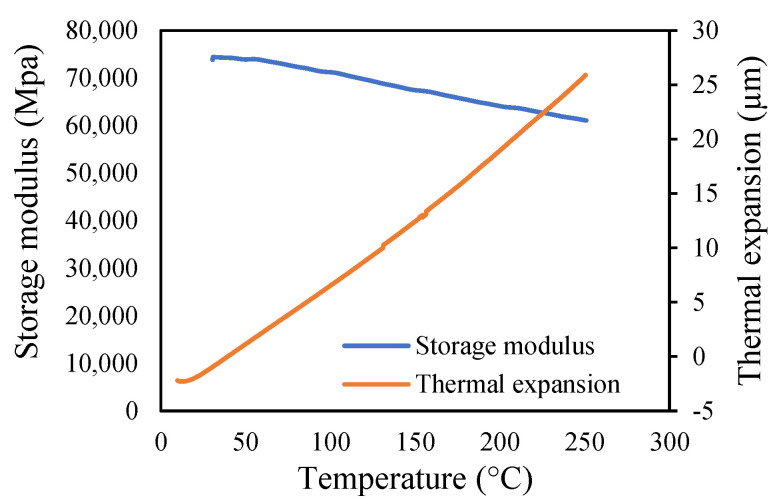
Thermal properties at different temperatures.

**Figure 28:**
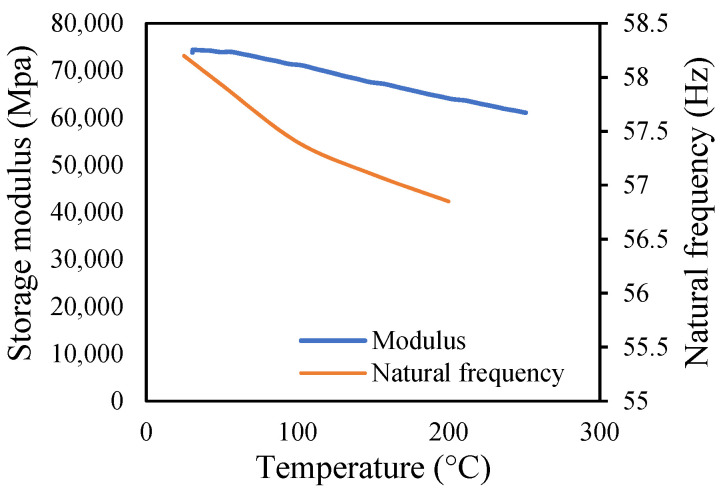
Modulus and frequency at different temperatures.

**Figure 29:**
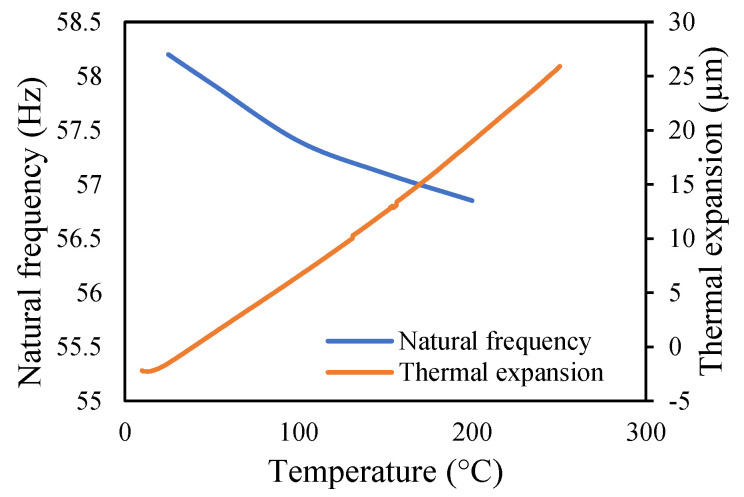
Thermal expansion and modal frequency at different temperatures.

The variations in the amplitude are due to the differences in the frequencies at the respective temperature range. The modal amplitude of uncracked and cracked beams is presented in Figure 23, Figure 24, Figure 25 and Figure 26. The modal parameters of the cantilever beam were examined with the changes in thermal properties due to the changes in temperatures. The illustration of the thermal properties concerning temperatures is shown in Figure 27. As is seen from the graph, the storage modulus decreases with an increase in temperature, while the coefficient of thermal expansion increases linearly with the elevation in temperature. The corresponding changes in natural frequencies with the changes in storage modulus are presented in Figure 28. It is seen that the fundamental frequencies of the cantilever beam are related to the changes in the modulus under given temperatures. However, the beam frequencies and thermal expansion are contrasting. The beam response decreases while thermal expansion increases when the temperatures rises, as shown in Figure 29. Thus, the modal characteristic of the cantilever beam is dependent on the nature of the changes in thermal properties concerning temperatures.

### 3.4. Crack Propagation Behavior When Subjected to Thermal and Mechanical Loads

**Figure 30:**
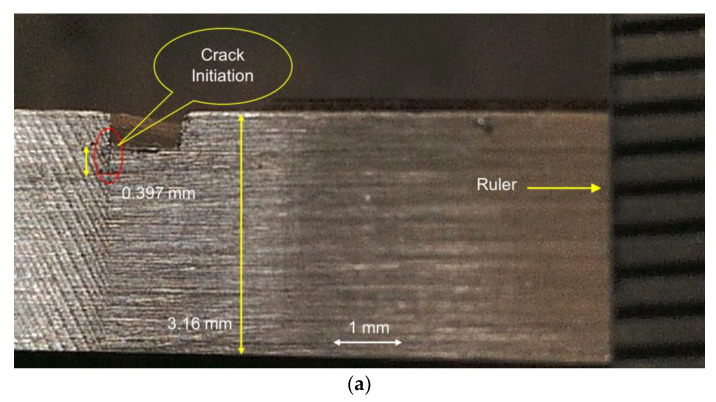

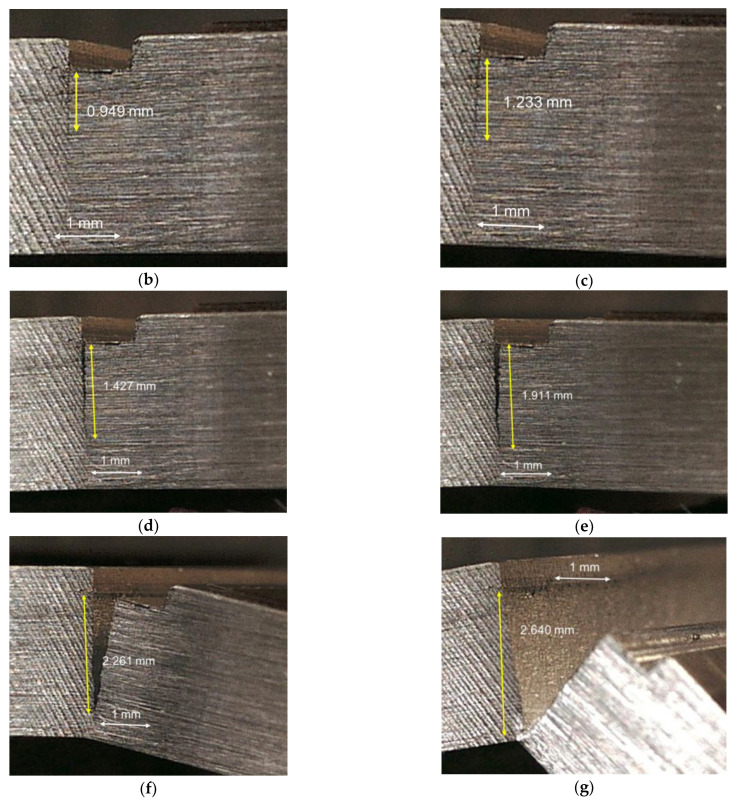
Sequence of crack propagation of pre-seeded 0.5 mm crack depth at room temperature. (**a**) 1st capture at crack initiation, (**b**) 2nd capture, (**c**) 3rd capture, (**d**) 4th capture, (**e**) 5th capture, (**f**) 6th capture, (**g**) 7th capture.

In this study, different types of pre-seeded crack specimens were chosen to analyze crack propagation. Crack depths of 0.2, 0.5 and 1 mm were considered for the uniform temperature distribution between 25 and 200 °C, while crack depths of 1, 1.3 and 1.5 mm were chosen for the non-uniform temperature distribution. The differences in crack depth for both temperature types were considered, because lower crack depth takes a longer time to propagate, which becomes a problem regarding maintaining the non-uniform temperature. Therefore, greater crack depths were considered under non-uniform analysis. The propagated crack depth was captured using a Dino-Lite Digital Microscope. Then, crack depths were measured based on pixels contained in the picture capture from the specimen. The propagated crack depth measurement for a pre-seeded crack depth of 0.5 mm at 25 °C is shown in Figure 30.

It was observed that crack propagation was quicker at lower temperatures than at elevated temperatures. This is due to the formation of plasticity at lower temperatures and more ductility at higher temperatures. It was also noticed that sometimes, a propagated crack develops multiple crack fronts within the pre-seeded crack. This is more frequent during the lower-temperature experiments. Moreover, the fatigue crack propagation was quicker when there was a single crack in the same direction. However, the propagation appeared sluggish whenever there was a dual crack front from the opposite end. Nevertheless, the speed of fatigue crack propagation was dependent on applied temperatures, attached end mass, and its alignment.

**Figure 31:**
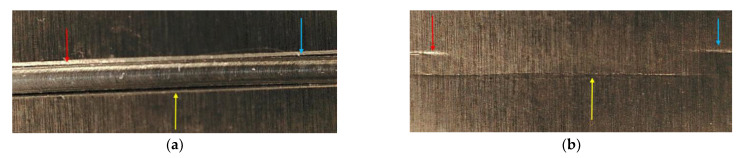

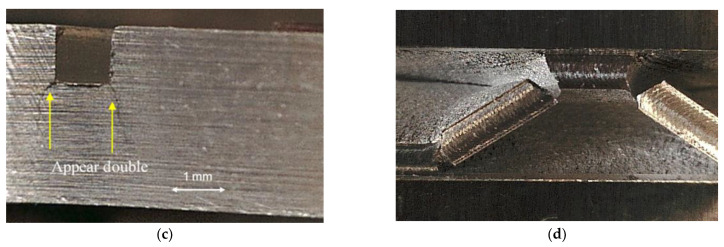
Double crack appearance in pre-seeded 1 mm crack depth. (**a**) Top view, (**b**) bottom view, (**c**) side view, (**d**) final breaking.

The evaluation of fatigue crack propagation at different temperatures showed a non-uniform crack pattern. This was, however, due to the selected shape of the pre-seeded crack. The occurrence of the first crack from the pre-seeded crack was not identical, even under the same boundary conditions. In this research, a rectangular-shaped pre-seeded crack was considered, which sometimes generated multiple cracks, as shown in Figure 31. It sometimes presented a double crack front. This propagation accelerates at lower temperatures than at elevated temperatures. This happened due to the growth of plasticity at the crack tip. Therefore, the modal parameters of the beam were not identical in the case of propagated crack. The actual crack and pre-seeded crack might be the problem. Hence, fatigue crack propagation evaluation on a pre-seeded crack, especially a rectangular crack, was not viable. This suggested that crack propagation analysis should be based on a real crack, or something resembling it.

Fatigue crack propagation evaluation was performed for various pre-seeded crack depths. The analysis was performed separately for uniform temperatures and non-uniform temperatures. The dynamic response of the beam was recorded during the crack propagation. The crack depth measurement and its modal parameters for a 0.25 mm pre-seeded crack depth subjected to isothermal and mechanical loads is given in Table 1. ’Total pixel’ is the total number of pixels contains in the specimen thickness. ’Scaled pixel’ is the number of pixels contained in a 1 mm ruler. Therefore, the number of pixels contained in 1 mm can be found. The crack depth pixel is the total number of pixels present in the crack. Hence, crack depth can be found by using the number of pixels contained in the crack. These procedures for measuring crack depth were repeated for each crack depth until total damage was achieved. It was observed that crack propagation was different for different temperatures. The corresponding natural frequency and amplitude were recorded during the crack propagation. It showed that the experimental results were comparable to the analytical results, although there was a small variation.

**Figure 32:**
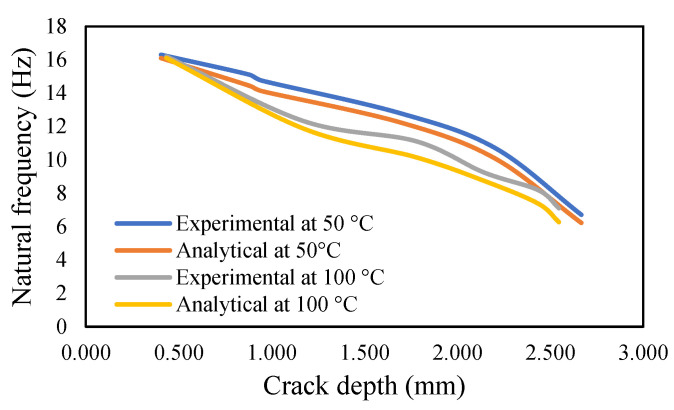
Comparison of the natural frequency of the propagated crack at an isothermal temperature of 50 and 100 °C.

**Figure 33:**
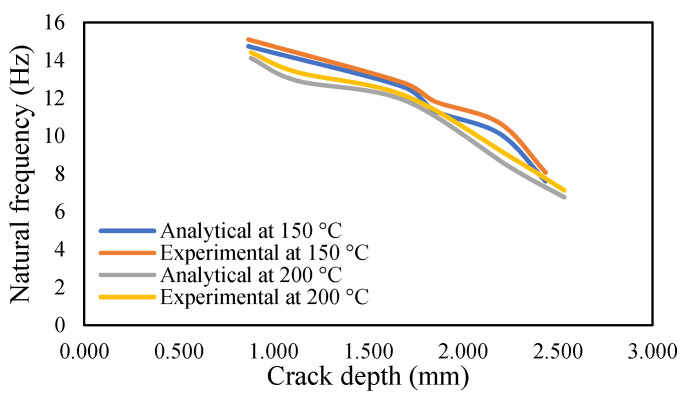
Comparison of the natural frequency of the propagated crack at an isothermal temperature of 150 and 200 °C.

**Figure 34:**
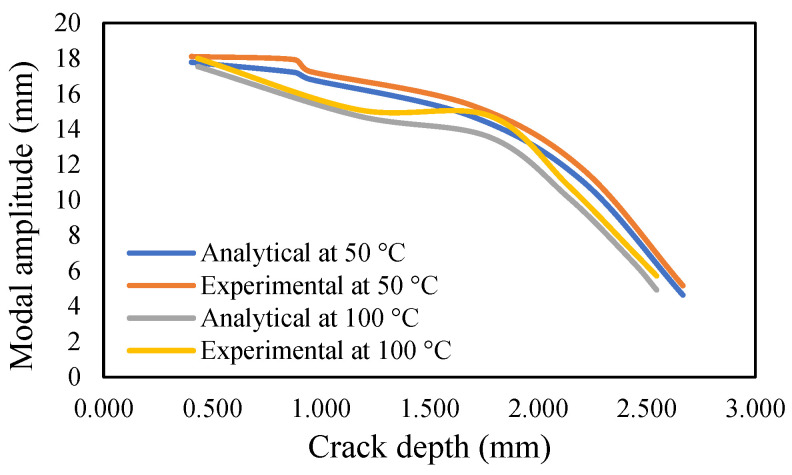
Comparison of the modal amplitude of the propagated crack at an isothermal temperature of 50 and 100 °C.

**Figure 35:**
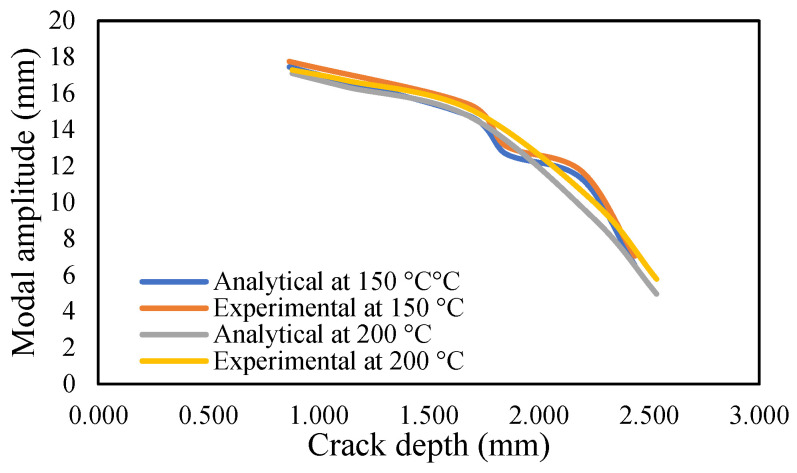
Comparison of the modal amplitude of the propagated crack at an isothermal temperature of 150 and 200 °C.

**Figure 36:**
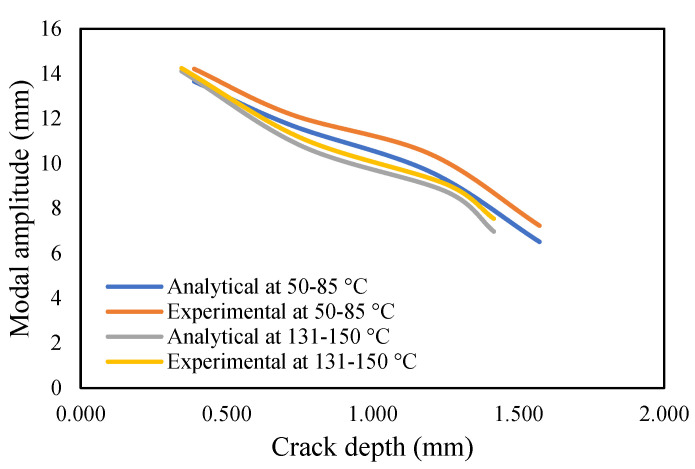
Comparison of the modal amplitude of propagated crack at a non-uniform temperature.

**Figure 37:**
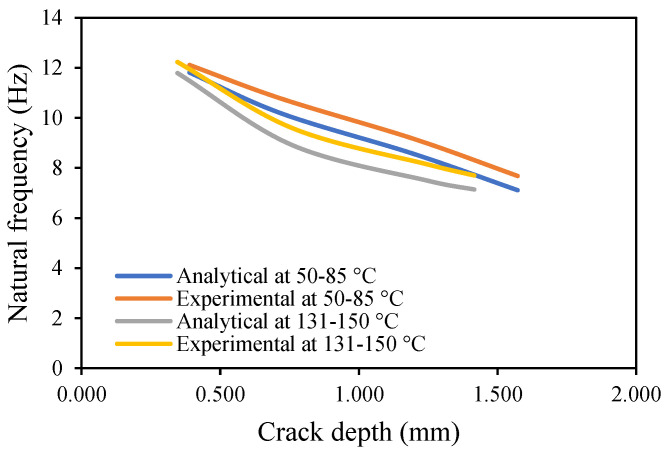
Comparison of the natural frequency of the propagated crack at a non-uniform temperature.

The results of crack propagation analysis show that a constant crack depth cannot be obtained for different temperatures. The propagation was not identical. The appearance of the first crack or crack initiation consumed much of the time in the whole propagation leading to failure. Additionally, the crack initiation was dependent on the pre-seeded crack depth. It was noticed that a deeper crack depth tends to appear to initiate cracking easily. Once the crack appeared, it was easier to accelerate the crack growth. This study observed that crack propagations were irregular, and they varied according to applied temperatures and pre-seeded cracks, as shown in the figures below. The comparison of frequency for isothermal temperatures are presented in Figure 32 and Figure 33, while Figure 34 and Figure 35 show the results of the modal amplitudes. The modal amplitudes are dependent on their fundamental frequencies; therefore, they vary with the variance in frequency. The comparison of modal parameters under non-uniform temperatures is presented in Figure 36 and Figure 37. The results show that modal parameters are more linear in nature than those of uniform temperatures. This suggests that the applied temperatures affect the modal parameters of the beam.

## 4. Conclusions

The vibration and fatigue cracking depending on structural dynamics are of serious concern in engineering applications. The applied temperature and heating rate can affect the overall structural integrity. This research aims to study the impact of applied thermal load and the heating rate on modal dynamics and crack propagation behavior. The evaluation showed that there was a variation in modal responses when changing the applied temperature, although the difference was small but considerable at high temperatures. The modal parameters were different for different heating rates, especially at elevated temperatures. This showed that experimental analysis at slow heating rates provided more precise results with the analytical results. The dissimilarities of modal response were also associated with the changes in thermal properties due to the change in temperatures.

This study demonstrated the fatigue crack propagation behavior of a cantilever beam under uniform and non-uniform temperatures conditions. The results of crack propagation demonstrate nonlinear crack growth at both high and low temperatures. Sometimes, double crack fronts and multiple cracks developed on the pre-seeded crack surface. However, this may be due to the selected pre-seeded crack shape. A pre-seeded rectangular crack shape was considered for this research. Hence, fatigue crack propagation evaluation on a pre-seeded crack, especially a rectangular crack, was not viable. It is worthwhile to choose a real crack for crack propagation analysis in the future.

## Figures and Tables

**Table 1:** Crack propagation at isothermal temperature of 50 °C for pre-seeded 0.25 mm crack depth.

Crack Depth (mm)	Crack Depth Pixel	Total Pixel	Scaled Pixel	1 mm = Pixel	1 Pixel = mm	Natural Frequency (Hz)		Modal Amplitude (mm)	
						Experiment	Analytical	Experiment	Analytical
0.4047	26	203	64	64.2405	0.0156	16.3	16.23	18.11	18.13
0.8786	57	205	65	64.8734	0.0154	15.11	14.97	17.93	17.85
0.9511	62	206	65	65.1899	0.0153	14.74	14.62	17.26	17.14
1.6874	110	206	65	65.1899	0.0153	12.81	12.65	15.38	15.21
2.1996	142	204	65	64.5570	0.0155	10.73	10.61	11.81	11.69
2.6667	173	205	64	64.8734	0.0154	6.71	6.68	5.16	5.13
2.8812	186	204	65	64.5570	0.0155	5.21	5.1	4.1	3.97

## Data Availability

The data presented in this study are available on request from the corresponding author.
